# Has China’s Pilot Emissions Trading Scheme Influenced the Carbon Intensity of Output?

**DOI:** 10.3390/ijerph16101854

**Published:** 2019-05-25

**Authors:** Kangkang Zhang, Deyi Xu, Shiran Li, Na Zhou, Jinhui Xiong

**Affiliations:** 1 Research Center of Resource and Environment Economics, Mineral Resource Strategy and Policy Research Center, China University of Geosciences, Wuhan 430074, China; zkk@cug.edu.cn (K.Z.); nazhou@cug.edu.cn (N.Z.); huijin0115@sina.com (J.X.); 2 School of Economics and Management, China University of Geosciences, Wuhan 430074, China; l2018sr@163.com

**Keywords:** ETS, carbon emission intensity, industry, generalized synthetic control method, China

## Abstract

China launched the pilot construction of the carbon emission trading scheme (ETS) in 2011. The pilots have been running for many years. Does ETS significantly restrain the increase of carbon emission intensity? Based on China’s panel data for provinces and industries, this paper uses the policy assessment method to evaluate the inhibition by ETS of carbon emission intensity. The assessment scope includes six provincial pilots and pilot industries covered by ETS. The results show that ETS has significant suppression of carbon emission intensity only in Beijing and Guangdong. There is no significant impact on the carbon emission intensity of Tianjin, Shanghai, Chongqing, and Hubei. Through the carbon emission intensity inhibition analysis of the industries covered by ETS from Beijing and Chongqing, the results of the production and supply of electric power, steam and hot water, petroleum processing and coking in Beijing have a significant impact on the ETS. Only the smelting and pressing of ferrous metals in Chongqing has a significant impact on the ETS.

## 1. Introduction

Since the 1990s, global warming and climate change have occurred with increasing greenhouse gas emissions [1,2,3]. Ecological environmental protection has once again become the focus of attention, and the carbon emission has become a major global problem [4,5,6,7]. In order to reduce the carbon emission, countries around the world have introduced the carbon emission reduction policies suitable for their own countries. The emission trading based on Coase’s first theorem has been put into practice and succeeded in many regions of the world, which is recognized as the most effective carbon emission reduction. The EU Emissions Trading System (EU-ETS), for example, which has been in operation since 2005, is the world’s largest and most mature ETS. After the end of the first phase, EU-ETS has gradually matured, which is an effective and efficient carbon emission reduction policy [8,9]. The famous Clean Air Amendment Act of the United States established a sulfur dioxide ETS and achieved great success. It is a glorious page in the history of emissions trading practice [10].

Since the beginning of the 21st century China’s industrialization and urbanization have accelerated, and the rapid economic development has led to a sharp increase in carbon emission. According to World Bank data, China’s carbon emission per capita in 2000 was 2.697 tons, but in 2011 reached 7.242 tons, an increase of nearly three times. China accounted for 28% of the global carbon emission in 2013 and became the world’s largest carbon emitter [11]. As the largest emitter of greenhouse gases of world, China has become a key player in the battle against global climate change [12]. Among them, China set and announced the mid-term quantitative greenhouse gas mitigation targets for 2020 and 2030 at the Copenhagen and Paris climate conferences. In order to control carbon emissions and achieve carbon emission reduction targets, the Chinese government has introduced a series of carbon emission reduction policies. In October 2011, the National Development and Reform Commission issued the Notice on the Implementation of Carbon Emission Trading Pilots, which approved the pilot projects of ETS in Beijing, Shanghai, Tianjin, Chongqing, Hubei, Guangdong, and Shenzhen. Between 2013 and 2014, the ETS market had been successively listed in these seven ETS pilots and the ETS markets began to operate formally. As of September 2017, the seven ETS pilots had integrated more than 20 industries and nearly 3000 key emission units. The cumulative transaction quota was about 197 million tons of carbon dioxide equivalent, and the cumulative turnover was about 4.516 billion yuan. The national ETS market was launched on December 19, 2017, and it is expected to build the world’s largest carbon emission trading market by 2020.

As an important way to reduce the carbon emission, ETS can accelerate the conversion of the carbon emission generated by economic entities into virtual carbon assets, which is to optimize the allocation of carbon assets through the market mechanism to reduce carbon emissions [13]. The pilots of China’s ETS have been running for many years. Does ETS play a significant carbon reduction effect? At the same time, due to regional differences, there are significant differences in the formulation of market entry rules for each pilot. How to formulate reasonable industry entry policies has attracted great attention from academic and political circles [14].

As early as the 1960s, the feasibility of property rights instruments for the control of gas emission pollution had attracted the attention of scholars. It is generally believed that the pollution emission trading method based on Coase’s first theorem will have a positive effect on reducing greenhouse gas emission. A large number of scholars had verified the necessity of constructing an emission trading market for developed and developing countries [15], and verified the impact between carbon emission reduction and GDP. Garbaccio et al. [16] found that developing countries implementing ETS-led emission reduction measures would significantly reduce the carbon emission. Hübler et al. [17] found that when the ETS market was established to achieve a 45% reduction commitment, the loss of GDP could be controlled at a level of 5%. With the continuous development of mathematical models and measurement tools, people began to purposefully conduct empirical research on the effectiveness of emission reductions established by ETS.

In the study of the effectiveness of ETS in carbon emission reduction, scholars mainly through the quotas [3,14,18,19,20,21], distribution methods [15,22,23,24], and carbon prices [13,14,25,26,27] in an ETS and other aspects of the analysis of the effectiveness of carbon reduction and its influencing factors [28,29]. The study found that the successful operation of an ETS may require a broader field of political and economic reform in China [30]. Although the implementation of an ETS has achieved initial results, the market efficiency of ETS pilots are not satisfactory [31]. Liu believed that China’s ETS market faced challenges, such as a lack of function, inaccurate quota allocation, imperfect trading mechanism, and lagging legislation. China’s ETS market has defects, such as no real-time carbon price and spot trading, and there are large differences with the functional system. The rapid integration of China’s ETS seems to be still far away [28]. Duan et al. showed that the coordination between the ETS and other policies were urgently needed in many aspects but, for various reasons, there were mostly deficiencies in practice, the most important of which may be the vested interests in the system [32].

By analyzing the existing literatures, it is found in the literature on the evaluation of the ETS, most of them analyzed from the perspectives of quotas, distribution methods and carbon prices, and there is a lack of analysis of the actual carbon emission intensity inhibition effect of the provinces. In addition to using the Computable General Equilibrium (CGE) model [12] to simulate the efficiency of sector coverage for the ETS, there are little discussions of the carbon emission reduction efficiency of sectors except of the power industry [33,34]. In view of the weak areas mentioned above, this paper will study the carbon emission intensity inhibition effectiveness of the ETS pilots based on the policy evaluation method, and measure the impact of ETS pilot construction on the carbon emission intensity of the region from a macro perspective. This paper also takes the industries covered by the ETS as the research object, analyzes the influence of carbon emission trading system on the carbon emission intensity of industries, and provides reference for the decision of the ETS market coverage.

In the literatures on the effectiveness of the ETS, scholars mainly study the impacts of the ETS on the carbon emission based on three models: the first is based on historical greenhouse gas emissions data, based on different scenarios for future greenhouse gas emissions. Forecasting and using this as a basis for evaluating the time-sequence model of ETS market emission reduction effects [10]. Secondly, using the difference-in-difference (DID) model to calculate and compare the carbon emission changes in ETS markets inside and outside the ETS mechanism to determine whether the ETS is effective [21,35]. The third is a CGE model which is used to predict the emission reduction effect of the ETS mechanism by setting different scenarios of total carbon emission management [12,36]. In above these models, the time series model and the CGE model belong to the pre-assessment method, and the DID model belongs to the post-evaluation method. Through literature analysis, it is found that in the aspect of model selection, the post-evaluation method mostly chooses the DID method, but the scientific nature of the DID method evaluation conclusion depends largely on the data quality and sample selection. The use of the DID method requires very strict conditions: the selection of the control group must be random, and the treatment group and control group need to be affected by common factors, and at the same development level, the data of the control group after the event must be stable. Since policy-makers do not make random decisions and economic growth in different regions are affected by geography, culture, economics, politics, et al., these conditions are difficult to meet in reality.

In order to improve the accuracy of policy evaluation, Abadie et al. [37,38], focused to expand and improve on the DID method, and proposed the Synthetic Control Method (SCM). In the selection process of the control group, the members of the optimal control group are selected, and the selected optimal control group is used to fit and predict the target data. However, this method does not make full use of the data, and, at the same time, the fitting level is not high due to the limitation of structure selection during the fitting process, and the result error is large. In order to make up for this deficiency, Xu [39] extended the SCM using the factor model, and obtained the Generalized Synthetic Control Method (GSCM), which constructs the factor variable by using all the information of the control group and the predictor variables of the target group. The factor load matrix of the factor variable is calculated according to the regression relationship between the factor variable and the target group data and, finally, the target group data is fitted. This method improves the fitting level and the credibility of the prediction. According to the data structures, this article uses the GSCM in the policy evaluation methods to study the impact of the implementation of ETS on the carbon emission intensity of the pilots and industries covered by the ETS. It not only describes in detail the effectiveness of carbon emission intensity suppression in pilots and industries, but also flexibly uses an effective assessment method to improve the accuracy and science of the results.

In this paper, the effects of six provincial pilot ETS markets on carbon emission intensity were evaluated using GSCM, the effects of ETS implementation on provincial carbon emission intensity were studied, and the industry conditions of the two ETS effects were analyzed. There are two innovative points in this study. First, the inhibitory effect of ETS on carbon emission intensity of pilot industries and industries covered by ETS is analyzed from a macro perspective. Second, the impact of ETS is more effectively analyzed using the latest policy assessment methods.

## 2. Scope and Methods

### 2.1. Research Scope

At the end of 2011, China launched a total of seven ETS pilots in two provinces and five cities. On the basis of industry emission level, emission reduction potential and pilot historical emission level, each pilot had formulated ETS market rules in accordance with the national total control principle and the ETS market segment of the seven pilots. The rules are shown in Table 1.

**Figure 1:**
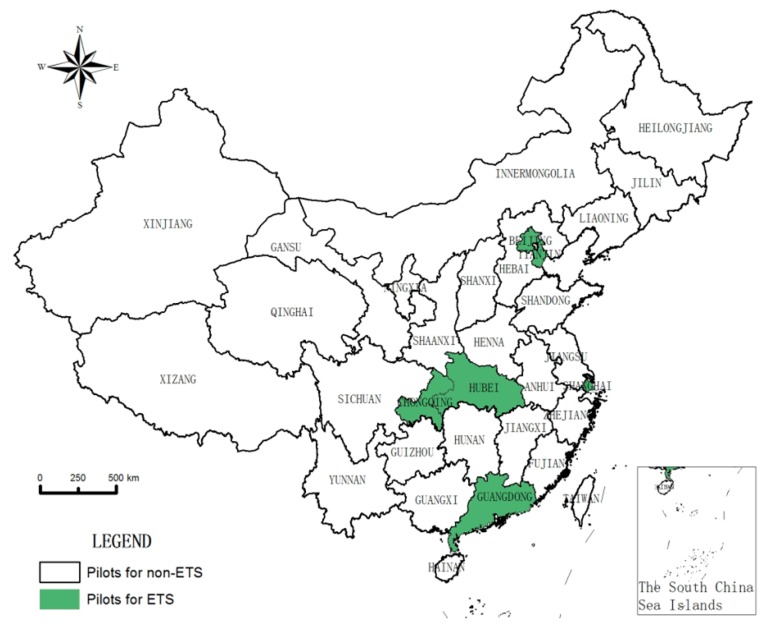
Pilot distribution of six provincial ETS markets in China.

Since Shenzhen is a municipal pilot and the control group is the provincial-level area, it is impossible to match Shenzhen and the control group. However, Shenzhen is affiliated to Guangdong Province, and the two pilots implemented an ETS in the same year, so this paper does not evaluate the effectiveness of the ETS in Shenzhen, but only considers the effectiveness of the ETS in Guangdong Province. The target group finally identified six pilots, namely Beijing, Shanghai, Tianjin, Chongqing, Hubei, and Guangdong, as shown in Figure 1. The other 24 provincial-level regions, except Hong Kong, Macao, Taiwan, and Tibet, will be used as the control group. Six provincial ETS pilots were launched at the end of 2011, but ETS markets were opened in 2013 and 2014, respectively, as shown in Table 1. The ETS market opening time in Beijing, Tianjin, Guangdong, and Shanghai was 2013, and the ETS market opening time in Chongqing and Hubei was 2014. When setting the time points for the establishment of each ETS market, the time of policy selection was selected as the time when the ETS market was opened. The ETS markets in Beijing, Tianjin, Guangdong, and Shanghai were established in 2013, and the establishment of ETS markets in Chongqing and Hubei were in 2014.

According to the scope of the emission reduction industry for each ETS pilot, each pilot covers different industries. In order to understand the industry’s emission reduction efficiency in the pilot and analyze the impact of industry contributions as effective or ineffective with respect to the ETS, this part is based on estimating the carbon emission intensity impact of various industries under the industrial category. According to the accessibility, representativeness, and comparability of the data, 32 industries were selected as the data for this study. Among them, Beijing selected the time from 2005–2016 and Chongqing selected the time from 2006–2016, as shown in Table 2.

### 2.2. Model Structure and Parameters

In recent years, under the background of national energy conservation and emission reduction, all provinces, cities, and industries have adopted extremely strict energy conservation and environmental protection policies, which have achieved fruitful carbon emission reduction results. China’s carbon emission per capita have been greatly reduced, and the carbon emission per capita in 2011–2017 have only increased from 7.242 tons to 7.554 tons. Due to the significant differences between China’s provinces, cities, and industries, the stability of the control group data is often affected by other policies and regional differences, which violate the assumption of the control group’s state invariance. It is difficult to evaluate the effectiveness of ETS. In order to solve this problem, we propose an assumption, as shown in **Assumption 1**.

**Assumption** **1.** *The influence of other policies related to carbon emission intensity is similar across China.*

According to the structural features of the available data, this paper selects the GSCM as the fitting model. The GSCM is commonly used in policy evaluation, and the effectiveness of policy implementation is judged mainly based on the relationship between the treated value and the estimated value of the post-treatment group. In this study, if the estimated value is larger than the treated value, it indicates that the implementation of the policy suppresses the increase of carbon emission intensity, and the policy formulation is effective. In other cases, the ETS does not curb the carbon intensity and the policy fails.

Assuming there are J+1 regions, and only the region *i* implements ETS, the rest of the regions are not affected by ETS. So we can use the remaining regions as control groups. Assume that the region *i* implements ETS at the time point of $t=T_{0}$, given the carbon emission intensity and other control group data for the regions J+1 during the period of t∈[1,T]. Let $y_{it}^{1}$ represent the carbon emission intensity data of region *i* implementing ETS at the time point $T_{0}$. Let $y_{it}^{0}$ represent the carbon emission intensity data of region *i* without the implementation of ETS at the period of $\left[1,T_{0}-1\right]$, and the carbon emission intensity of the regions is not affected by the implementation of ETS in the period, that is,$y_{it}^{1}=y_{it}^{0}$. After the implementation of ETS in the period of $\left[T_{0},T\right]$, let $\delta_{it}=y_{it}^{1}-y_{it}^{0}$ represent the treatment effect of the implementation of ETS in region *i* on the carbon emission intensity at the time point *t*.

For the regions where ETS is implemented, we can observe its carbon emission intensity data $y_{it}^{1}$ during the period of $t\in [T_{0},T]$, but we cannot observe the carbon emission intensity data $y_{it}^{0}$ after ETS in these region. Therefore, this article uses the GSCM to estimate the carbon emission intensity estimates $y_{it}^{0}$ in the regions where ETS is implemented during the period of $t\in [T_{0},T]$. As shown in Equation (1):

(1) $$y_{it}^{0}=x_{it}^{\prime }\beta +\lambda_{i}^{\prime }f_{t}+\varepsilon_{it}$$

Among them, $x_{it}$ is the covariate of the (k×1) dimension, that is the control variable. $\beta ={\left[\beta_{1},\ldots ,\beta_{k}\right]}^{\prime }$ is the common factor of the (k×1) dimension unobservable, which is the dimension unknown parameter. $f_{t}={\left[f_{1t},\ldots ,f_{rt}\right]}^{\prime }$ is a (r×1) dimensional unobservable common factor. $\varepsilon_{it}$ is an unobservable short-term shock with an average of zero at the regional level.

The calculation formula of average treatment effect (ATT) of carbon emission intensity is shown in Equation (2):

(2) $$ATT_{it}=y_{it}^{1}-y_{it}^{0}$$

where $y_{it}^{1}$ is the treated value of the dependent variable for policy implementation and $y_{it}^{0}$ is the estimated value of the dependent variable for which the policy is not implemented.

### 2.3. Data and Variables

According to the theme of this article, the carbon emission intensity, which is calculated from the ratio of the carbon emission to the development level (GDP or industrial gross output value), is selected as the target variable. Since there are no official statistics on the carbon emissions of provinces and industries, this paper selects the carbon emissions calculated from the energy consumption data over the years as the target variable. According to China Energy Statistical Yearbook 2017, specific conversion methods are given to calculate carbon emissions. Combined with the background of the research object, nine types of energy consumption categories are finally selected as the basis for calculation of the carbon emission, namely coal, gasoline, diesel, natural gas, kerosene, fuel oil, crude oil (liquefied petroleum gas), coke, and electricity. Among them, when calculating the carbon emission data of various provinces, cities and industries, the total amount of the nine types of energy consumption is converted into standard coal and multiplied by the respective carbon emission coefficient. The carbon emission calculation formula is as shown in Equation (3):

(3) $$C_{it}=\Sigma (A_{ijt}\times E_{j}\times \eta_{j})$$

where $C_{it}$ is the total carbon emission in region *i* in the time *t*. And $A_{ijt}$ is the energy consumption in time t in region *i*. $E_{j}$ is the standard statistic when energy *j* is converted to standard coal. $\eta_{j}$ is the carbon emission coefficient of energy type *j*. The energy carbon emission coefficient is shown in Table 3.

According to the availability of data, combined with Equation (3) and Table 3, carbon emission data of 30 provinces and 32 industries in China, except Hong Kong, Macao, Taiwan, and Tibet, were calculated (energy data in the industry data does not include crude oil data, but does include liquefied petroleum gas data). In order to improve the fitting level of GSCM, exogenous variables are added as predictor variables. Consider the availability and integrity of data, and refer to the research results of relevant literatures on the analysis of carbon emission intensity and carbon emission influencing factors [52,53,54], the final predictor variables selected in the fitting model of provincial carbon emission intensity, including opening up (OU), means the ratio of total import and export trade to GDP. Population factor (POP) represents the total population of the region at the end of the year. Fixed assets investment (FAI) represents the fixed added value of each province over the years. Economic development level (EDL) represents gross domestic product data and is adjusted for constant prices based on 1997. Industrial structure (IS) represents the ratio of the sum of the secondary industry and the tertiary industry to the gross domestic product of the region. The total value of household consumption (CPI) represents the total value of household consumption in each province in the past year. Seven factors that mainly affect carbon emission intensity are taken as predictor variables. The predictor variables of the fitting model of industry carbon emission intensity include average wage (AW) represents the average wage of employees. Employment (EM) refers to the average number of employees. Industrial added value (IDV) represents the added value of industrial enterprises above a certain size. It is assumed that these factors are not affected by the ETS.

The data above come from China National Bureau of Statistics, China Economic and Trade Network Statistics Database, China Economic and Trade Network Industry Database, and the EPS Data Platform. In order to eliminate the problems of heteroscedasticity, etc., this paper performs logarithmic processing on the data, and the logarithmically processed data are stable through the robustness test.

## 3. Results

### 3.1. The Impact of ETS on Carbon Emission Intensity in Provincial Pilots

**Figure 2:**
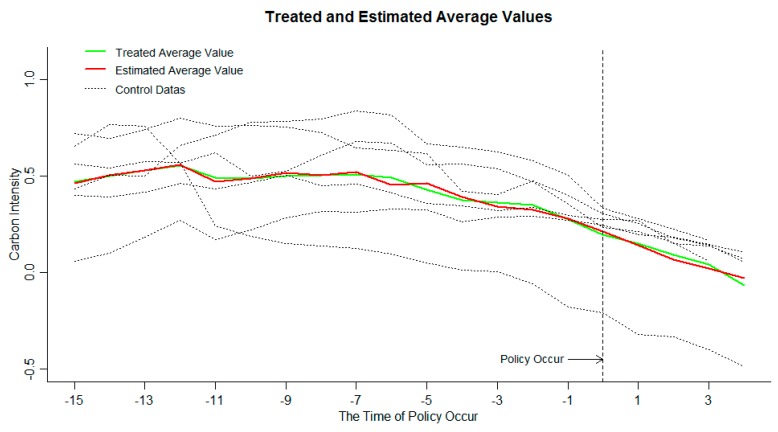
Treated values and estimated values carbon emission intensity of the target group.

Due to the inconsistent start-up time of ETS, this article evaluates the effectiveness of each pilot’s ETS. Among them, Beijing, Shanghai, Tianjin, and Guangdong started in 2013, using 2013–2016 data as the time period for evaluating policy effects. Chongqing and Hubei’s ETS markets were established in 2014 and the data during 2014–2016 were used as a time frame for assessing policy effects. According to the theoretical principle of the GSCM, if the treated value is less than the estimated value during the time period of $T\geq T_{0}$, it indicates that the establishment of ETS has a significant effect on the inhibition of carbon emission intensity. If the treated value is larger than the estimated value, or approximates the estimated value, it shows that ETS did not achieve the effect of suppressing carbon emission intensity, and the policy failed. The carbon emission intensity of each pilot and the mean data are shown in Figure 2. The carbon emission intensity is treated logarithmically.

The carbon emission intensity and the average value of the six provincial ETS pilots have been significantly reduced, indicating that the carbon emission intensity in the pilot areas has been decreasing year by year, and the emission reduction policies have significant effects. From the relationship between the treated values and the estimated values of the target group in Figure 2, it can be seen that in the early stage of ETS, the actual mean value of carbon emission intensity and the estimated mean value change trend are synchronous and basically coincide, indicating that the GSCM has a high degree of fit to the processing group data. It can be seen from the data at the end of the policy that the two lines are close together and appear to be alternate. It cannot be considered that ETS has a significant inhibitory effect on carbon emission intensity, and the implementation of ETS fails. This paper believes that there are two possible reasons for this result:

First, the ETS in some regions has a significant inhibitory effect on carbon emission intensity, however, there is no significant inhibitory effect in other areas, and carbon emission intensity goes up. When calculating the mean value, positive and negative values offset each other, resulting in an insignificant reduction in the mean value of the overall carbon emission intensity.

Second, the ETS has no significant impact on the carbon emission intensity of each pilot; the treatment effect of carbon emission intensity in each pilot is zero.

**Figure 3:**
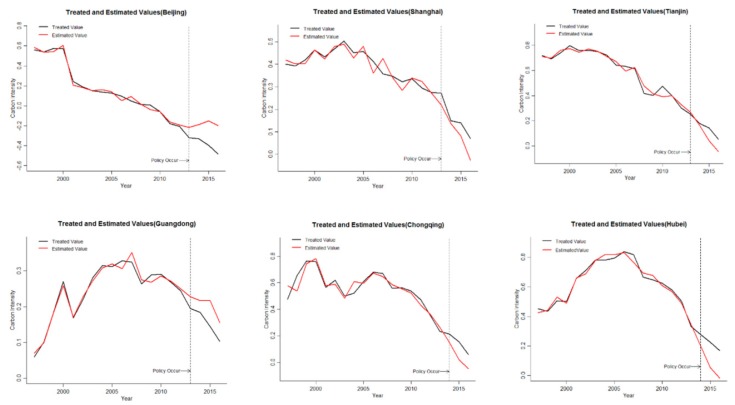
The treated value and the estimated value of the pilots.

In order to analyze the carbon emission intensity inhibition effect of ETS of each provincial pilot, this part evaluates the effectiveness of ETS on carbon emission intensity suppression in six provincial pilots. The carbon emission intensity is treated logarithmically. The results are shown in Figure 3.

ETS has been significantly effective in suppressing carbon emission intensity in Beijing and Guangdong, and has no effect on carbon emission intensity inhibition in other regions. It can be seen from Figure 3 that the left side of the dotted line is the time period before the ETS. According to the relationship between the treated value of the carbon emission intensity in the target area and the estimated value, the numerical trend is consistent and the values are basically coincident, indicating that the method can fit well to the actual carbon emission intensity changes of each pilot, and the fitting effect is better. In the period after the implementation of ETS, the estimated value of Beijing and Guangdong are larger than the treated value, indicating that ETS has effectively reduced the carbon emission intensity in Beijing and Guangdong, and the ETS pilots can be active and effective for carbon emission intensity. By comparing the size of the difference, it is found that the difference of Beijing is obviously larger than that of Guangdong. The effect of ETS on Beijing’s carbon intensity was stronger than that of Guangdong. The estimated value in Tianjin, Shanghai, Chongqing, and Hubei are less than the treated value, ETS has no effective restraining effect on carbon emission intensity. ETS in four pilot areas were ineffective, contrary to ETS’s original design.

ETS in Beijing and Guangdong is significantly effective in suppressing carbon emission intensity. However, the data for Guangdong is the result of two pilots in Guangdong and Shenzhen, Shenzhen is the market with the largest number of enterprises among the seven pilot areas, and the region with a smaller total quota in the seven pilot areas. As a result, the market demand is large and the trading frequency is frequent, resulting in the hottest ETS phenomenon in Shenzhen. However, due to the lack of city-level data, it is impossible to analyze the ETS in Shenzhen, so it is impossible to judge which ETS in Guangdong and Shenzhen has a significant carbon emission reduction. The ETS in Beijing has a significant inhibitory effect on carbon emission intensity, and the inhibitory effect is more effective over time.

### 3.2. The Impact of ETS on the Carbon Emission in Industries

According to the above analysis results, the carbon emission intensity of the six provincial pilots is affected by an ETS in two ways, that is, the implementation of an ETS has a significant impact on the carbon emission reduction of Beijing and Guangdong, but has no significant impact on the carbon emission reduction of the other four regions. To further analyze the effective carbon reductions of industries covered by an ETS, this part analyzes the effectiveness of the industry’s carbon reduction impacts from the implementation of an ETS based on an industry perspective. This part selects industries covered by an ETS in Beijing and Chongqing. The reasons for selecting industries in Beijing and Chongqing for effectiveness evaluation are as follows: Beijing and Chongqing are both municipalities directly under the central government and are consistent in the attributes of the administrative units. From the analysis results, the implementation of ETS in Beijing has played a significant role in carbon emission reduction, but the impact in Chongqing is not significant. Thus, in order to further analyze the carbon emission reduction of the industry, the impact of the ETS is very necessary for analysis in different regions. Although Tianjin is also a municipality directly under the central government, the implementation of the ETS in Tianjin has no significant impact on carbon emission reduction since Tianjin is adjacent to Beijing, and Beijing, Tianjin, and Hebei belong to the same economic circle. There are close links in policy and management. The analysis of Tianjin may be affected by Beijing. Therefore, this part selects the analysis of the effectiveness of carbon emission reduction in Beijing and Chongqing.

**Figure 4:**
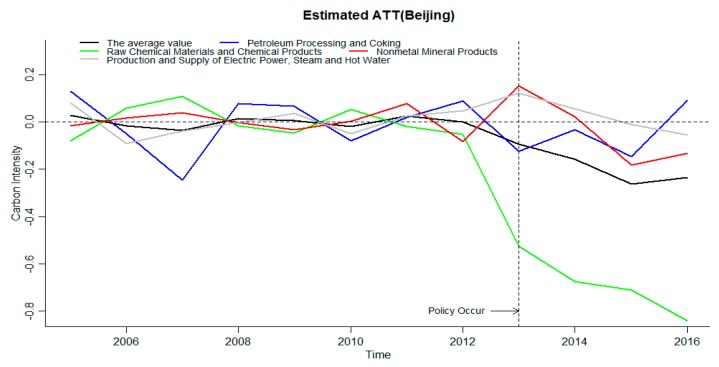
The impact of an ETS on industry carbon emission reduction rates in Beijing.

**Figure 5:**
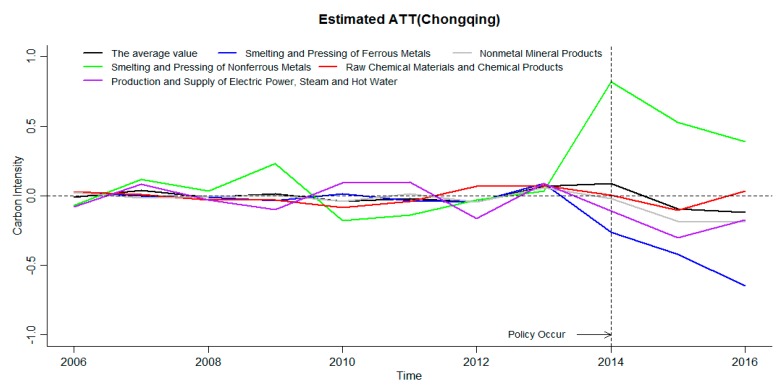
The impact of an ETS on industry carbon emission reduction rates in Chongqing.

As shown in Table 1, the ETS in Beijing started in November 2013, and the ETS in Chongqing started in June 2014. The time range for the treatment effect of carbon emission reduction intensity in this part is the time range for implementing the ETS, that is, the assessment scope of Beijing is 2013–2016, and the assessment scope of Chongqing is 2014–2016. The average treatment efficiency (ATT) of carbon emissions of industries covered by the ETS in Beijing and Chongqing is shown in Figure 4 and Figure 5, where the estimated ATT is the difference between the treated value and the estimated value. The carbon emission intensity is treated logarithmically.

ETS can significantly reduce the average carbon intensity of ETS industries in Beijing, and increase the carbon intensity of Petroleum processing and coking and the raw chemical materials and chemical products. The carbon emission intensity of the nonmetal mineral products and the production and supply of electric power, steam and hot water is significantly inhibited in the later stage. As can be seen from Figure 4, all values are around zero before the policy occurs, indicating that the GSCM can be used to fit the data very significantly, and providing support for the reliability of the results of the processing effect after the policy occurs. The average value of the carbon emission reduction treatment effects of the four industries is shown in the black line in Figure 4. When the policy occurs, the treatment effect average is less than zero, and it shows a downward trend year by year, the ETS has a significant inhibitory effect on the carbon emission intensity. Additionally, the intensity of influence increases year by year. The treatment effects of the petroleum processing and coking and the raw chemical materials and chemical products were all below zero after the policy occurred, and the ETS significantly inhibited the increase in carbon emission intensity of the industries. The treatment effect of the nonmetal mineral products and the production and supply of electric power, steam and hot water is greater than zero after the implementation of ETS, but it is reduced by less than zero in the later stage, and the ETS has no significant impact on the carbon emission intensity of the nonmetal mineral products and the production and supply of electric power, steam and hot water in the current period, but the impact is significant in the later period, which inhibits the increase of carbon emission intensity. Through the analysis of the impact of the ETS in four industries covered by the ETS in Beijing, the ETS significantly inhibited the treatment effect of carbon emission intensity in four industries, and the average value of four industries also decreased significantly, the ETS having a significant inhibitory effect on carbon emission intensity of industries in Beijing. The absolute value of the raw chemical materials and chemical products is the largest and increases year by year. The ETS has the strongest inhibitory effect on the raw chemical materials and chemical products carbon emission intensity in Beijing, and the trend is increasing.

The inhibitory effect of the ETS on the smelting and pressing of nonferrous metals in Chongqing is not significant, and the other four industries have significantly suppressed the increase in carbon emission intensity. It can be seen from Figure 5 that before the policy occurrence time, the processing effects are all around zero, the policy evaluation method based on the GSCM is also significantly effective in analyzing the data. The carbon emission intensity is treated logarithmically. Among them, the average of the carbon emission intensity treatment effects of the five industries covered by the ETS is greater than zero in the current period of ETS implementation, but significantly less than zero after the implementation of the ETS. The average carbon emission intensity of the five industries has no significant inhibitory effect on the carbon emission intensity when the ETS is implemented. However, the average treatment effect after the implementation is less than 0. The ETS can significantly inhibit the increase of carbon emission intensity of the covered industries in Chongqing. In the analysis of five industries, it is found that the ETS has no significant effect on the carbon emission intensity of the smelting and pressing of nonferrous metals, but has significant effect on the carbon emission intensity of other four industries. The absolute value of the smelting and pressing of ferrous metals is the highest and increases year by year. The implementation of ETS has the strongest inhibitory effect on the carbon emission intensity of the smelting and pressing of ferrous metals in Chongqing. Based on the above analysis, it is found that one of the reasons why ETS’s overall carbon emission inhibition effect is not significant in Chongqing is caused by the abnormality of individual industries. Based on the analysis of the emission reduction effectiveness of the covered industries, it can be seen that the effective emission reduction coverage of the industry in Chongqing is significantly stronger than that in Beijing.

## 4. Robustness Test

ETS pilots were carried out under the guidance of the Notice on Piloting ETS issued by the National Development and Reform Commission in October 2011. In order to better operate an ETS, all pilots will discuss and formulate corresponding carbon emission reduction policies during the preparation stage of ETS market listing, so as to promote the effective emission reduction of carbon emission by the ETS. Therefore, whether an ETS has significant inhibitory effect on carbon emission intensity, that is, whether the inhibitory effect of carbon emission intensity, is due to the influence of an ETS. Based on this, this part examines the changes in the timing of the ETS with reference to the idea of placebo testing. Assuming that the ETS is not running at the real start time, the above method is used to analyze the policy and compare the policy effect at the real start time to analyze the effectiveness of ETS implementation. The test time is selected before and after the actual start of the ETS. According to the test results, the above analysis results are judged to be robust. The timeliness judgment criteria is shown in Table 4.

### 4.1. Timeliness Test of ETS in Carbon Trading in Pilot Provinces

**Figure 6:**
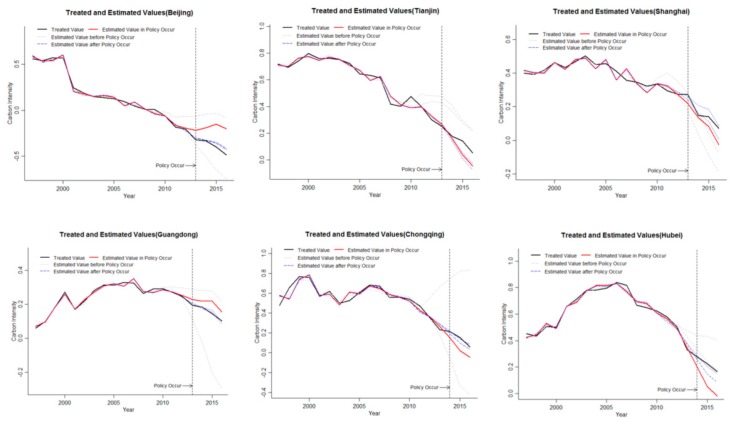
Carbon emission reduction effect robustness test.

The time-effectiveness test results of carbon emission intensity inhibition effect in provincial pilots are shown in Figure 6, and the carbon emission intensity is treated logarithmically.

It can be seen from Figure 6, combined with the inspection rules in Table 4, only two regions, Beijing and Guangdong, passed the test, and ETS can effectively curb the carbon emission intensity in Beijing and Guangdong. The other four regions, including Tianjin, etc., failed the validity test. The effect of ETS in Tianjin and other regions on carbon emission intensity is not significant

### 4.2. Timeliness Test of ETS in Carbon Trading in Industries

**Figure 7:**
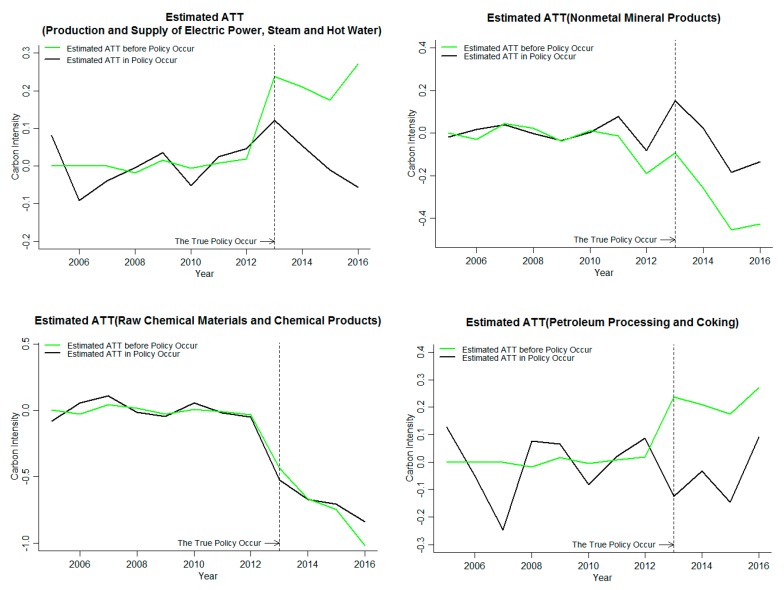
Industry robustness test results in Beijing.

**Figure 8:**
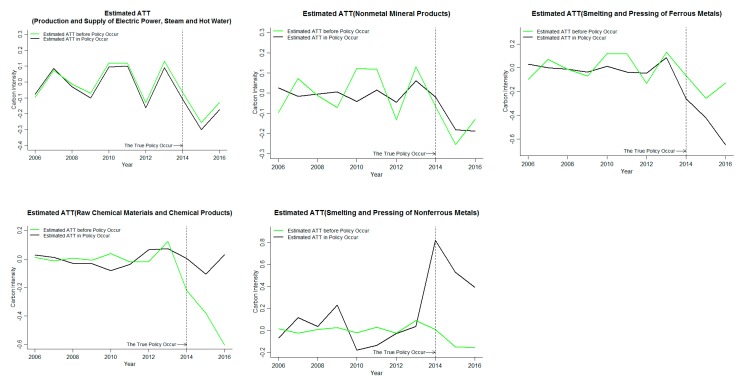
Industry robustness test results in Chongqing.

With the above inspection principle, the timeliness of the industries covered by ETS in Beijing and Chongqing will be tested. The inspection time is one year before the implementation of ETS. The robustness test time point in Beijing is 2012, and the robustness test time point in Chongqing is 2013. The carbon emission intensity is treated logarithmically. The test results are shown in Figure 7 and Figure 8.

The results of the two industries, namely, the production and the supply of electric power, steam and hot water and the petroleum processing and coking in the industries covered by ETS in Beijing, passed the robustness test. Combined with the judgment criteria of Table 4, it can be seen from the results of the robustness test of Figure 7 that the data before the policy of the supply of electric power, steam and hot water and the petroleum processing and coking is significantly larger than the actual data at the time of policy occurrence. The implementation of the ETS can significantly inhibit the carbon emission intensity of the industries. However, the effect on carbon emission intensity of the nonmetal mineral products and the raw chemical materials and chemical products is not significant.

Among the industries covered by the carbon market in Chongqing, only the results of the smelting and pressing of ferrous metals is significant, and other industries cannot pass the robustness test. Combined with the judgment criteria of Table 4, through the robustness test of the industry, it is found that the estimated value before the policy of the smelting and pressing of ferrous metals is greater than the estimated value of the actual occurrence point of the policy. The rest of the industries are unable to pass the robustness test significantly.

Through the robustness test of industries covered by the carbon market in Beijing and Chongqing, the results of the production and supply of electric power, steam and hot water and petroleum processing and coking in Beijing passed the robustness test. Only the smelting and pressing of ferrous metals in Chongqing has a remarkable test of robustness. The impact of the ETS on the industries in Beijing is significantly larger than that in Chongqing. Therefore, combining the above the provincial carbon emission intensity of the analysis results shows that the effects of ETS, relative to Beijing, one of the important reasons for the insignificant effect of the ETS on carbon emission intensity in Chongqing is that few industries covered by the ETS have effective emission reduction.

## 5. Discussion

According to the results of this paper, it can be found that the implementation of ETS does not have a significant inhibitory effect on the carbon emission intensity of all pilots and industries. With the national carbon market fully open by the end of 2017, how to design reasonable and effective ETS guidelines is a problem faced by the government and scholars. Through the research of this paper, we know that only two of the six provincial pilots have achieved the expected inhibitory effect. By analyzing the effectiveness of industry emission reduction, it is found that only two of the industries covered by an ETS in Beijing pass the robustness test. The carbon emission reduction effect of ETS in various pilots and industries in China is very unsatisfactory. If the national ETS is established according to the pilot policies, the result will not meet the expectations of policy-makers. Therefore, ETS in China cannot effectively curb the carbon emission intensity, and its effect on environmental governance is not significant. This paper only analyzes the effectiveness of ETS implementation from the perspective of overall carbon emission intensity. The results are not decisive enough to provide a detailed reference for the construction of a national carbon market, but can provide a reference for the government to supervise the carbon market and help the government to understand the emission reduction situation of the carbon market.

Although the central government has the right of direct leadership over the governments of various provinces and cities, there are relatively free regulatory systems in various provinces and cities, which are not completely subject to the overall restriction of the central government. All provinces and cities formulate appropriate emission reduction policies according to their own characteristics. Therefore, the study in this paper has limitations. Not only may other strategies for controlling carbon emission intensity be different, but there may be other important omitted variables. Each region will formulate different emission reduction policies according to its economic development, regional characteristics and pollution level. For example, as Beijing is the capital city of China, the pollution control strategies include the relocation of polluting industries, etc., while Shanghai vigorously develops the tertiary industry. Therefore, the assumption in this paper may lack sufficient conditions to be fully established in this context. Other neglected variables also affect the robustness of the results. For example, in the validity analysis of the provincial carbon emission intensity implemented by ETS in this paper, the control variables include opening up (OU), population factor (POP), fixed assets investment (FAI), economic growth (GDP), industrial structure (IS), etc., and there are still other important variables that make the fitting effect better. The existence of these limitations may lead to the deviation of the conclusions of this paper, but the pilot study and method of this paper have reference value for future research and the construction of a national ETS in China.

## 6. Conclusions

The implementation of ETS in various pilots in China does not all inhibit carbon emissions, mainly due to the lack of implementation of pilot enterprise participation, policy formulation, and carbon emission reduction in various industries. In view of China’s rapid industrial development and economic transformation, how to formulate a reasonable and effective ETS policy is the main task of establishing seven pilots. Through the carbon emission reduction effectiveness evaluation of carbon trading implementation, it is helpful for the government to understand the implementation effect of ETS in general, and provide a reference for the later active and in-depth analysis of its causes.

In this paper, the GSCM is used to evaluate the effectiveness of ETS on carbon emission intensity reduction in six provincial pilots and industries covered by an ETS. It is found that an ETS has significant suppression of carbon emission intensity only in Beijing and Guangdong. The effect on carbon intensity of Tianjin, Shanghai, Chongqing, and Hubei was not significant.

In order to analyze the impact of ETS on the carbon market coverage industries, the GSCM is used to analyze the carbon emission reduction effectiveness of the carbon market coverage industries in Beijing and Chongqing. It was found that the implementation of ETS can significantly inhibit the increase of the average carbon emission intensity of industries in Beijing, as well as the increase of carbon emission intensity of the petroleum processing and coking and the raw chemical materials and chemical products, the nonmetal mineral products and the production and supply of electric power, steam and hot water are significantly inhibited in the later stage. ETS did not significantly affect the smelting and pressing of nonferrous metals in Chongqing, but significantly reduced the carbon emission intensity of the remaining four industries. However, according to the robustness test results, the results of two industries covered by the carbon market in Beijing, namely, the production and supply of electric power, steam and hot water and the petroleum processing and coking, passed the robustness test. Among the industries covered by the carbon market in Chongqing, only the smelting and pressing of ferrous metals had significant results, and all other industries failed the robustness test.

## Figures and Tables

**Table 1:** Overview of the rules of ETS markets in the pilots.

ETS Pilots	Opening Time	Access Rules	Industry to Reduce Emissions	Mode of Doing Business	Quota Allocation Method	Quotas Issued Method
Shenzhen	2013-06-18	Enterprises that emit more than 20,000 tons, large public buildings of 0.2 million tons	Industry (electricity, water, manufacturing, etc.) and construction	Agreement transfer and Open auction	Historical emissions law and Baseline method	Free
Beijing	2013-11-28	The average emissions in 2009–2011 exceeded 10,000 tons	Industry: electricity, heat, cement, petrochemical, other industries and services	Agreement transfer and Open auction	Historical emissions law	Free
Tianjin	2013-12-26	Enterprises and civil buildings that have discharged more than 20,000 tons since 2009	5 industrial sectors: electric power, steel, chemical, petrochemical, oil and gas exploration and civil construction	Agreement transfer and Open auction	Historical emissions law and Baseline method	Free and Paid
Shanghai	2013-11-26	In 2010–2011, emissions exceeded 20,000 tons (industrial) 10,000 tons (non-industrial)	10 industrial sectors: electricity, steel, petrochemical, chemical, nonferrous metals, building materials, textiles, paper, rubber and chemical fiber 7 non-industrial industries: aviation, airports, ports, shopping malls, hotels, business office buildings and railway sites	Agreement transfer and Open auction	Historical emissions law and Baseline method	Free
Chongqing	2014-06-19	Emissions exceeding 20,000 tons or annual energy consumption exceeding 10,000 tons of standard coal	Electricity, electrolytic aluminum, ferroalloy, calcium carbide, caustic soda, cement, steel	Agreement transfer and Open auction	Historical emissions law and Baseline method	Free
Guangdong	2013-12-19	2011–2014 emissions exceeding 20,000 tons or energy consumption exceeding 10,000 tons of standard coal	4 industrial sectors: electricity, cement, steel, petrochemical After 2014, it expanded to other industrial industries as well as non-industrial complexes such as hotels, restaurants, finance, commerce, and public institutions	Agreement transfer and Open auction	Historical emissions law and Baseline method	Free and Paid
Hubei	2014-04-02	60,000 t standard coal energy consumption enterprise	12 industrial sectors: electricity and heat, non-ferrous metals, steel, chemicals, cement, petrochemicals, automotive glass, chemical fiber, paper, medicine, food and beverage	Open auction	Historical emissions law and Baseline method	Free

Source of data: Relevant policies and documents published in each pilots, as well as trading websites [40,41,42,43,44,45,46].

**Table 2:** Industry ID marking details.

ID	Industry	ID	Industry	ID	Industry
I01	Coal Mining and Dressing	I12	Furniture Manufacturing	I23	Metal Products
I02	Petroleum and Natural Gas Extraction	I13	Papermaking and Paper Products	I24	Ordinary Machinery
I03	Ferrous Metals Mining and Dressing	I14	Printing and Record Medium Reproduction	I25	Equipment for Special Purposes
I04	Nonferrous Metals Mining and Dressing	I15	Cultural, Educational and Sports Articles	I26	Electric Equipment and Machinery
I05	Nonmetal Minerals Mining and Dressing	I16	Petroleum Processing and Coking	I27	Electronic and Telecommunications Equipment
I06	Food Processing	I17	Raw Chemical Materials and Chemical Products	I28	Instruments, Meters, Cultural and Office Machinery
I07	Food Production	I18	Medical and Pharmaceutical Products	I29	Scrap and waste
I08	Textile Industry	I19	Chemical Fiber	I30	Production and Supply of Electric Power, Steam and Hot Water
I09	Garments and Other Fiber Products	I20	Nonmetal Mineral Products	I31	Production and Supply of Gas
I10	Leather, Furs, Down and Related Products	I21	Smelting and Pressing of Ferrous Metals	I32	Production and Supply of Tap Water
I11	Timber Processing, Bamboo, Cane, Palm Fiber & Straw Products	I22	Smelting and Pressing of Nonferrous Metals		

Source: EPS Data Platform [47] and Beijing and Chongqing Statistical Yearbook [48,49].

**Table 3:** Energy carbon emission factor.

Types of Energy	Standard Statistic (kg Standard Coal/kg)	Carbon Emission Coefficient (kg Carbon/kg Standard Coal)
Coal	0.7143	0.7559
Coke	0.9714	0.8550
Crude Oil	1.4286	0.5857
Gasoline	1.4714	0.5538
Kerosene	1.4714	0.5714
Diesel Oil	1.4571	0.5913
Fuel Oil	1.4286	0.6185
Natural Gas	1.33	0.4483
Electric Power	0.1229	2.2132
Liquefied Petroleum Gas	1.7143	0.5042

Among them, the standard statistical unit of natural gas is kg standard coal/m^3^. The standard unit of measurement of electricity is kg standard coal/kWh. Source: 2017 China Energy Statistical Yearbook [50] and related literature [51].

**Table 4:** Timeliness judgment criteria for ETS.

ETS Timeliness Test Standard for Carbon Emission Reduction	
Real time point pro phase < real time point ≤ real time point post phase	The result is effective
Others	The result is invalid
